# Metabolomics profiling reveals changes in peptidoglycan and redox metabolism between ancient and modern *Mycobacterium tuberculosis*

**DOI:** 10.1128/spectrum.02887-25

**Published:** 2026-06-29

**Authors:** Ashleigh Cheyne, Elisabeth V. Oskoui, Agnieszka Broda, Nitya Krishnan, Yi Liu, Brian D. Robertson, Myrsini Kaforou, Gerald Larrouy-Maumus

**Affiliations:** 1 Centre for Bacterial Resistance Biology, Department of Life Sciences, Faculty of Natural Sciences, Imperial College London 98992 , London, United Kingdom; 2 Department of Infectious Disease, Imperial College London 4615, London, United Kingdom; 3 Centre for Pediatrics and Child Health, Imperial College London 4615, London, United Kingdom; Tata Institute of Fundamental Research 29146, Mumbai, India

**Keywords:** tuberculosis, metabolomics, lineages

## Abstract

*Mycobacterium tuberculosis* (Mtb), the cause of tuberculosis, kills over 1 million people worldwide each year. Mtb is classified into 10 distinct lineages, each harboring unique genetic changes that influence its survival, virulence, and transmissibility. Although metabolism plays a critical role in Mtb infection and persistence, the metabolomic profiles of different Mtb lineages remain poorly characterized. Here, by using metabolomic and bioinformatic approaches, we have determined the metabolome of representative strains belonging to three main lineages, namely lineage 1, lineage 2, and lineage 4. We show that the ancient lineage 1 has considerable differences in its metabolome compared to lineages 2 and 4. Those differences are mainly related to amino acids, peptidoglycan synthesis intermediates, and ergothioneine, a sulfur-containing histidine derivative with potent antioxidant and redox properties. Taken together, these data represent the first comprehensive analysis of the metabolome of three main Mtb lineages.

**IMPORTANCE:**

Metabolic regulation of *Mycobacterium tuberculosis* (Mtb) underlies its successful infection and persistence. Previous metabolic characterization has largely been limited to the reference strain H37Rv, but there are significant metabolic differences between ancient and modern lineages. This study illustrates the complexity of metabolic regulation in Mtb and the limitations of using a single strain to develop understanding. Investigation of lineage-specific changes in Mtb metabolism could help identify weaknesses that could be therapeutically targeted and could inform the evaluation of broad-spectrum antibiotics.

## INTRODUCTION

Understanding the evolution of pathogens is vital to inform public health guidelines. The study of evolutionary differences between strains of the same pathogen can indicate biological pathways that are critical for increased fitness, virulence, and drug resistance. *Mycobacterium tuberculosis* (Mtb) has evolved over millennia to adapt to its human hosts (1), leading to the divergence of Mtb subspecies and lineages spread across the globe (2). There are currently 10 Mtb lineages that are recognized based on nucleotide variations (3–6). For example, some lineages predominate in specific geographical locations: lineage 1 in East Africa and South East Asia; lineage 3 in East Africa and Asia; lineages 5 and 6 in West Africa; and lineage 7 in Ethiopia (3). Other lineages are more dispersed, with lineage 2 found in both Southeast Asia and Europe, and lineage 4 found in Africa, America, South America, Asia, Australia, and Europe (6). Previous studies have focused on how host heterogeneity leads to differences in Mtb infection and disease outcomes; however, research over the last decade has also identified differences among Mtb lineages themselves, such as in their genome (7), lipidome (8), and virulence (9). These lineage-specific differences are reflected in the host response to infection. For example, Krishnan et al. (10) found that strains from lineages 1 and 2 induce a significantly higher anti-inflammatory response compared to strains in lineage 4, stimulated by bacterial lipids.

Recent studies have shown that single nucleotide variations in Mtb strains often result in differences in virulence, antibiotic resistance, and immune response in the host (11–16). However, it remains poorly understood whether lineage-specific genetic variants alter downstream metabolic pathways (17).

Here, we present a comprehensive metabolomics analysis reporting on metabolites analyzed by liquid chromatography-mass spectrometry (LC-MS) across three major Mtb lineages, 1, 2, and 4. Our approach revealed that compared to the reference strain Mtb H37Rv and lineages 2 and 4, ancient lineage 1 strains showed a change in their metabolome which could contribute to the reduced ability of lineage 1 strains to adapt to host stresses (18–20).

## MATERIALS AND METHODS

### *Mycobacterium tuberculosis* strains and growth conditions

A list of Mtb strains can be found in Table 1. Mycobacterial strains were grown to mid-exponential phase in 7H9 liquid medium supplemented with 0.5 g/L Fraction V bovine serum albumin, 0.05% tyloxapol, 0.2% dextrose, 0.2% glycerol, and 10 mM NaCl. Throughout the study, mycobacteria were cultured in a shaking incubator set at 125 rpm and 37°C. The doubling time was calculated as described (21).

**TABLE 1:** Mycobacterial strains used in this study

Strain	Spoligotype^*a*^	Lineage^*b*^	Origin of acquisition	Origin of patient sample	Database	Sample accession	Genome accession
Mtb H37Rv	N/A	Lineage 4 (EA)	Laboratory collection	N/A^*c*^	NCBI	ASM19595v2	GCA_00195955
Mtb 232	EA14_VNM	Lineage 1 (IO)	Krishnan et al. (10)	Vietnam	ENA	SAMEA1877123	ERR234238
Mtb 281	ZERO	Lineage 1 (IO)	Krishnan et al. (10)	Vietnam	ENA	SAMEA1877076	ERR234207
Mtb 346	EA14_VNM	Lineage 1 (IO)	Krishnan et al. (10)	Vietnam	ENA	SAMEA1877209	ERR234240
Mtb 372	EA14_VNM	Lineage 1 (IO)	Krishnan et al. (10)	Vietnam	ENA	SAMEA1877222	ERR234241
Mtb 119	BEIJING	Lineage 2 (BJ)	Krishnan et al. (10)	Vietnam	ENA	SAMEA1877286	ERR234193
Mtb 212	BEIJING	Lineage 2 (BJ)	Krishnan et al. (10)	Vietnam	ENA	SAMEA1877267	ERR234190
Mtb 345	BEIJING-LIKE	Lineage 2 (BJ)	Krishnan et al. (10)	Vietnam	ENA	SAMEA1877289	ERR234209
Mtb 374	BEIJING	Lineage 2 (BJ)	Krishnan et al. (10)	Vietnam	ENA	SAMEA1877242	ERR234242
Mtb 649	BEIJING-LIKE	Lineage 2 (BJ)	Krishnan et al. (10)	Vietnam	ENA	SAMEA1877261	ERR234211
Mtb 173	H1	Lineage 4 (EA)	Krishnan et al. (10)	Vietnam	ENA	SAMEA1877245	ERR234170
Mtb 293	T1	Lineage 4 (EA)	Krishnan et al. (10)	Vietnam	ENA	SAMEA1877074	ERR234180
Mtb 318	T1	Lineage 4 (EA)	Krishnan et al. (10)	Vietnam	ENA	SAMEA1877128	ERR234239
Mtb 440	H3	Lineage 4 (EA)	Krishnan et al. (10)	Vietnam	ENA	SAMEA1877184	ERR234243
Mtb 639	H3	Lineage 4 (EA)	Krishnan et al. (10)	Vietnam	ENA	SAMEA1877200	ERR234244

^*a*^ Classified according to the International Spoligotyping Database (22).

^*b*^ BJ, East Asian/Beijing; EA, Euro/American; O, Indo/Oceanic. NCBI stands for National Center for Biotechnology Information. ENA stands for European Nucleotide Archive. The database and sample accession code can be used to access the respective genetic sequence files. The project code in ENA is PRJEB3223.

^*c*^ N/A, not applicable.

### Metabolite extraction

For the metabolomics profiling studies, experiments were performed in two biological and three technical replicates. First, the Mtb strains were initially grown in 7H9 liquid medium containing the carbon sources of interest until the OD_600_ reached 0.8–1. One microliter of bacteria was then loaded onto 0.22 μm nitrocellulose filters under vacuum filtration. The mycobacteria-laden filters were then placed on top of chemically equivalent agar medium (described above) and allowed to grow at 37°C for five doubling times to generate sufficient biomass for targeted metabolomics studies. The bacteria were metabolically quenched by plunging the filters into extraction solution composed of acetonitrile/methanol/H_2_O (2:2:1) precooled to 4°C. Small molecules were extracted by mechanical lysis of the entire bacteria-containing solution with 0.1 mm acid-washed zirconia beads for 1 min using a FastPrep (MP Bio) set to 6.0 m/s. The lysates were filtered twice through 0.22 μm Spin-X column filters (CoStar). The bacterial biomass of the individual samples was determined by measuring the residual protein content of the metabolite extracts using the BCA assay kit (Thermo) (23, 24). A 100 μL aliquot of the metabolite solution was then mixed with 100 μL of acetonitrile with 0.2% acetic acid at −20°C and centrifuged for 10 min at 17,000 × *g* and 4°C. The final concentration of 70% acetonitrile was compatible with the starting conditions of chromatography. The supernatant was then transferred into LC-MS V-shaped vials (Agilent 5188-2788), and 4 μL was injected into the LC-MS instrument (Agilent 6545 QToF).

### Liquid chromatography-mass spectrometry for metabolomics profiling

Aqueous normal-phase liquid chromatography was performed using an Agilent 1290 Infinity II LC system equipped with a binary pump, temperature-controlled autosampler (set at 4°C) and temperature-controlled column compartment (set at 25°C) containing a Cogent Diamond Hydride Type C silica column (150 mm × 2.1 mm; dead volume of 315 µL). A flow rate of 0.4 mL/min was used. The elution of polar metabolites was performed using solvent A, which consisted of deionized water (resistivity ~18 MΩ cm) and 0.2% acetic acid, and solvent B, which consisted of 0.2% acetic acid in acetonitrile. The following gradient was used at a flow rate of 0.4 mL/min: 0 minute, 85% B; 0–2 min, 85% B; 3–5 min, 80% B; 6–7 min, 75% B; 8–9 min, 70% B; 10–11 min, 50% B; 11.1–14 min, 20% B; 14.1–25 min, 20% B; and 5-min re-equilibration period at 85% B. Accurate mass spectrometry was performed using an Agilent Accurate Mass 6545 QTOF apparatus. Dynamic mass axis calibration was achieved by continuous infusion after chromatography of a reference mass solution using an isocratic pump connected to an ESI ionization source operated in the positive-ion mode. The nozzle and fragmentor voltages were set to 2,000 and 100 V, respectively. The nebulizer pressure was set to 50 psig, and the nitrogen drying gas flow rate was set to 5 L/min. The drying gas temperature was maintained at 300°C. The MS acquisition rate was 1.5 spectra/s, and *m*/*z* data ranging from 50 to 1,200 were stored. This instrument enabled accurate mass spectral measurements with an error of less than 5 parts per million (ppm), a mass resolution ranging from 10,000 to 45,000 over the *m*/*z* range of 121–955 atomic mass units, and a 100,000-fold dynamic range with picomolar sensitivity. The data were collected in the centroid 4 GHz (extended dynamic range) mode. The detected *m*/*z* data were deemed to be metabolites identified based on unique accurate mass-retention time and MS/MS fragmentation identifiers for masses exhibiting the expected distribution of accompanying isotopomers. The typical variation in abundance for most of the metabolites remained between 5% and 10% under these experimental conditions.

### Metabolomics data analysis

Target list of metabolites (*n* = 43) related to central carbon metabolism and nitrogen synthesis pathways, including glycolysis, the TCA cycle, the urea cycle, peptidoglycan production, amino acids, and nucleoside synthesis, was created using Agilent MassHunter Pathways to PCDL B.08.00 software. The software generates a target personal compound database library (PCDL) that contains names and chemical formulas. Addition of metabolite retention times in the target PCDL was provided from a pre-existing in-house database. This was then used for targeted features extraction in Agilent MassHunter Profinder 8.0 sp3 (Tables S1 and S3). The metabolite data were normalized with log10 and Pareto scaling (mean centered and divided by the square root of the standard deviation) in MetaboAnalyst (v6.0) (25). In R, non-averaged strain sample values were used to calculate significant differences in metabolites between lineage groups with Welch’s *t*-test and (Benjamini-Hochberg) FDR-adjusted *P* values. Welch’s *t*-test, which does not assume equal variances, was chosen because groups had different numbers of strains. Normalized metabolite abundances were averaged over the two biological and three technical repeats per strain and then averaged over lineages to calculate log10 fold change (L10FC) for lineages 1, 2, and 4 from the reference strain H37Rv.

### Sequencing and single-nucleotide polymorphism (SNP) calling

All *Mycobacterium tuberculosis* (Mtb) strains were sequenced, and SNPs determined before the commencement of this current study. Sequencing was performed with Illumina on a HiScanSQ instrument to obtain reads between 50 and 100 base pairs, as previously described (1, 26). Each sequenced genome was checked with FASTQC and aligned to the annotated Mtb reference genome using BWA (27). SNP calling for each sample was performed with Samtools, filtered to include only those with a read depth above 7 and mapping quality above 50, and then annotated with SNPEff (27, 28). The annotated H37Rv genome can be accessed from the National Center for Biotechnology Information (NCBI) database and the other 14 strain genomes from the European Nucleotide Archive (ENA) database (Table 1).

### Metabolomic data visualization

The main metabolomics workflow code is available at GENMETA-TB on GitHub (https://github.com/evoskoui/GENMETA-TB).

All metabolites with a significant change from reference strain Mtb H37Rv (2 FC and <0.05 FDR) were included in the further analysis. KEGG IDs were assigned, if necessary, and then the KEGG database (29) was queried with the KEGGREST function in R (https://bioconductor.org/packages/KEGGREST) to select any Mtb pathways with those metabolites present. KEGG Mtb pathways were then queried to select any enzymes present. Pathways from the BioCyc database (Mtb enzymes SmartTable) (30) were connected to metabolites in a similar manner. Related genetic data were joined by the encoding gene code for each enzyme (Rv code), successfully matching metabolites to enzyme-encoding genes. The metabolic genes list was analyzed to determine if metabolic changes occurred alongside genetic changes in the same pathway and lineage. Pathways with significant metabolic changes were examined in more detail.

## RESULTS

### Non-synonymous SNPs differentiate Mtb strains by their lineages

**Fig 1:**
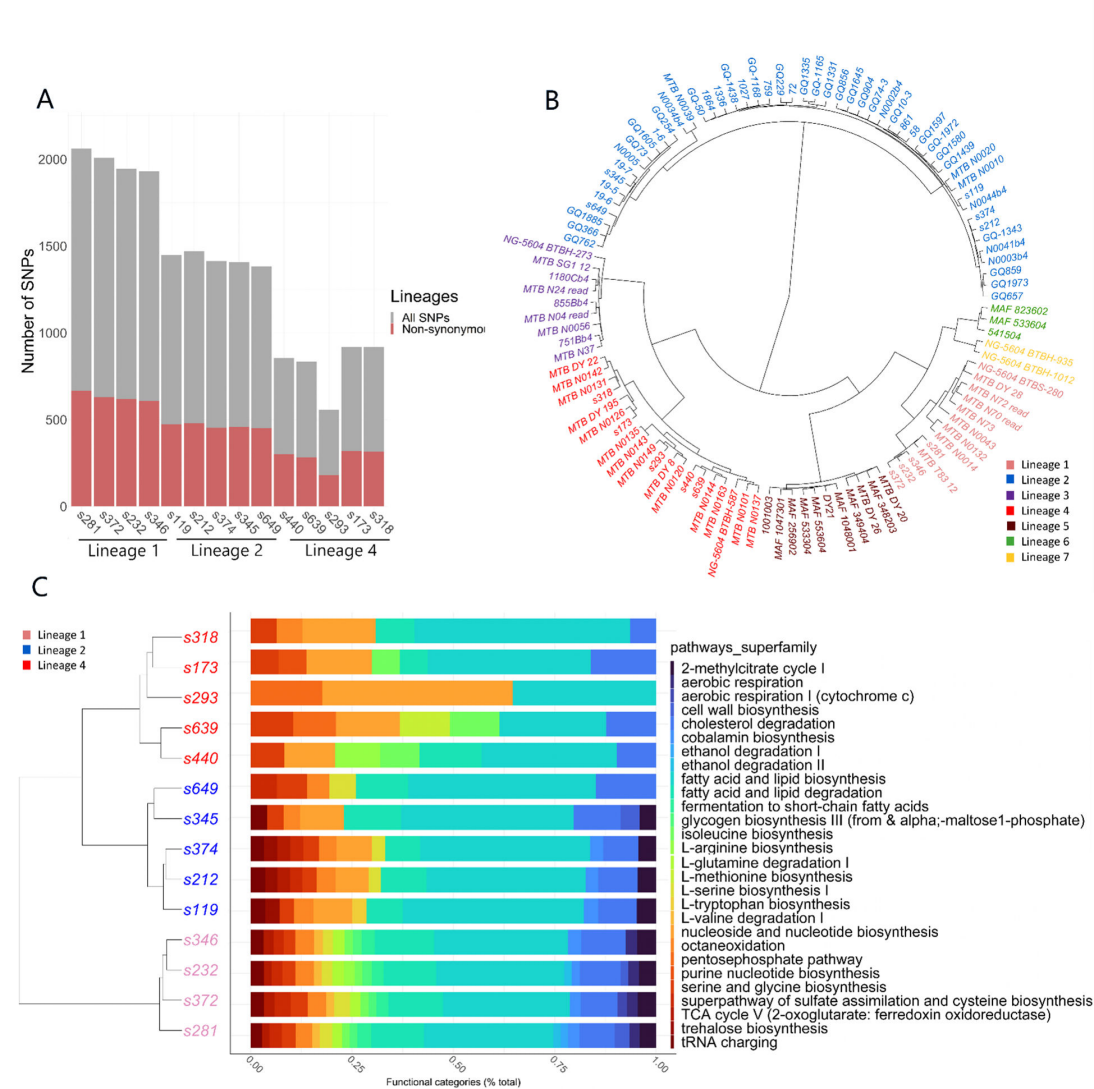
Non-synonymous SNPs cluster strains into lineage-specific categories and suggest evolution of genes affecting numerous metabolic pathways. (**A**) Total number of SNPs and non-synonymous SNPs identified in Mtb strains from lineages 1, 2, and 4. (**B**) Phylogenetic tree constructed from all SNPs of Vietnamese Mtb strains and 95 publicly available Mtb strains from lineages 1–7. (**C**) Dendrogram clustering based on non-synonymous SNPs and functional category annotation of genes targeted by non-synonymous SNPs using BioCyc. Annotations are displayed as a percentage of total functional categories affected by non-synonymous SNPs per strain.

In this study, we selected 14 strains from Vietnam from Mtb lineages 1, 2, and 4 (10). To confirm the genotypic classification of the Mtb strains by lineage, we compared the sequenced genomes of the selected strains against the Mtb H37Rv genome as a reference to detect SNPs. After filtering for a read depth of greater than 7 and a mapping quality greater than 50, a total of 5,258 SNPs were identified across all strains, of which 3,501 were non-synonymous. Lineage 4 showed the lowest number of SNPs compared to the reference strain H37Rv, followed by lineage 2, then lineage 1, which was expected as the reference strain is part of lineage 4 (Fig. 1A).

To illustrate how these strains fit into the Mtb phylogeny with other lineages, SNPs were analyzed from 95 Mtb strains available from public data sets representing lineages 1–7 (Fig. 1B). The 95 additional strains were processed using the same bioinformatics pipeline and reference genome as the 14 strains from Vietnam. The Vietnamese strains clustered alongside other strains of the same lineages in the global data set. These strains did separate within lineage groups, due to differences in spoligotypes between strains.

To determine the potential phenotypic impact of non-synonymous SNPs, we functionally categorized the genes affected by these SNPs using BioCyc categories (30). A total of 731 individual SNPs were identified in genes with unknown function and were therefore removed from analysis, alongside functional categories with fewer than 5 SNPs present. Of the remaining SNPs, mapping to 5,406 functional categories, clear differences between lineages were identified (Fig. 1C). Within lineage 4, SNPs were associated with pathways such as cholesterol degradation, fatty acid synthesis and degradation, and nucleoside and nucleotide biosynthesis. Lineage 2 also contained SNPs affecting the same pathways, and additional SNPs were found in genes in the 2-methylcitrate cycle, cobalamin biosynthesis, methionine biosynthesis, propanoyl-CoA degradation, serine and glycine biosynthesis, sulfate assimilation, and cysteine biosynthesis, the TCA cycle, and trehalose biosynthesis and rRNA charging. The SNPs found in lineage 1 strains affected the largest number of functional pathways, with SNPs in genes in all the previously mentioned pathways, plus additional SNPs in genes involved in cell wall biosynthesis, arginine biosynthesis, and glutamine degradation pathways.

### Metabolomics analysis of Mtb strains identifies lineage-specific differences in metabolomes

Our functional category analysis suggested significant metabolic remodeling in several pathways. To investigate whether these genome-level differences lead to downstream metabolic alterations, we first measured the bacterial growth doubling times for each strain. Differences in bacterial doubling times could be indicative of differences in the central carbon metabolism, which is required to sustain growth. However, no significant changes were observed in doubling time (Table 2).

**TABLE 2:** Doubling time of the strains used in this study

	Strain name	Doubling time (h)
Lineage 1	Mtb 232	26 ± 4
	Mtb 281	24 ± 5
	Mtb 346	25 ± 5
	Mtb 372	24 ± 3
Lineage 2	Mtb 119	20 ± 3
	Mtb 212	29 ± 4
	Mtb 345	34 ± 1
	Mtb 374	36 ± 5
	Mtb 649	30 ± 6
Lineage 4	Mtb 173	19 ± 3
	Mtb 293	25 ± 7
	Mtb 318	41 ± 11
	Mtb 440	27 ± 2
	Mtb 639	34 ± 7
	Mtb H37Rv	23 ± 4

**Fig 2:**
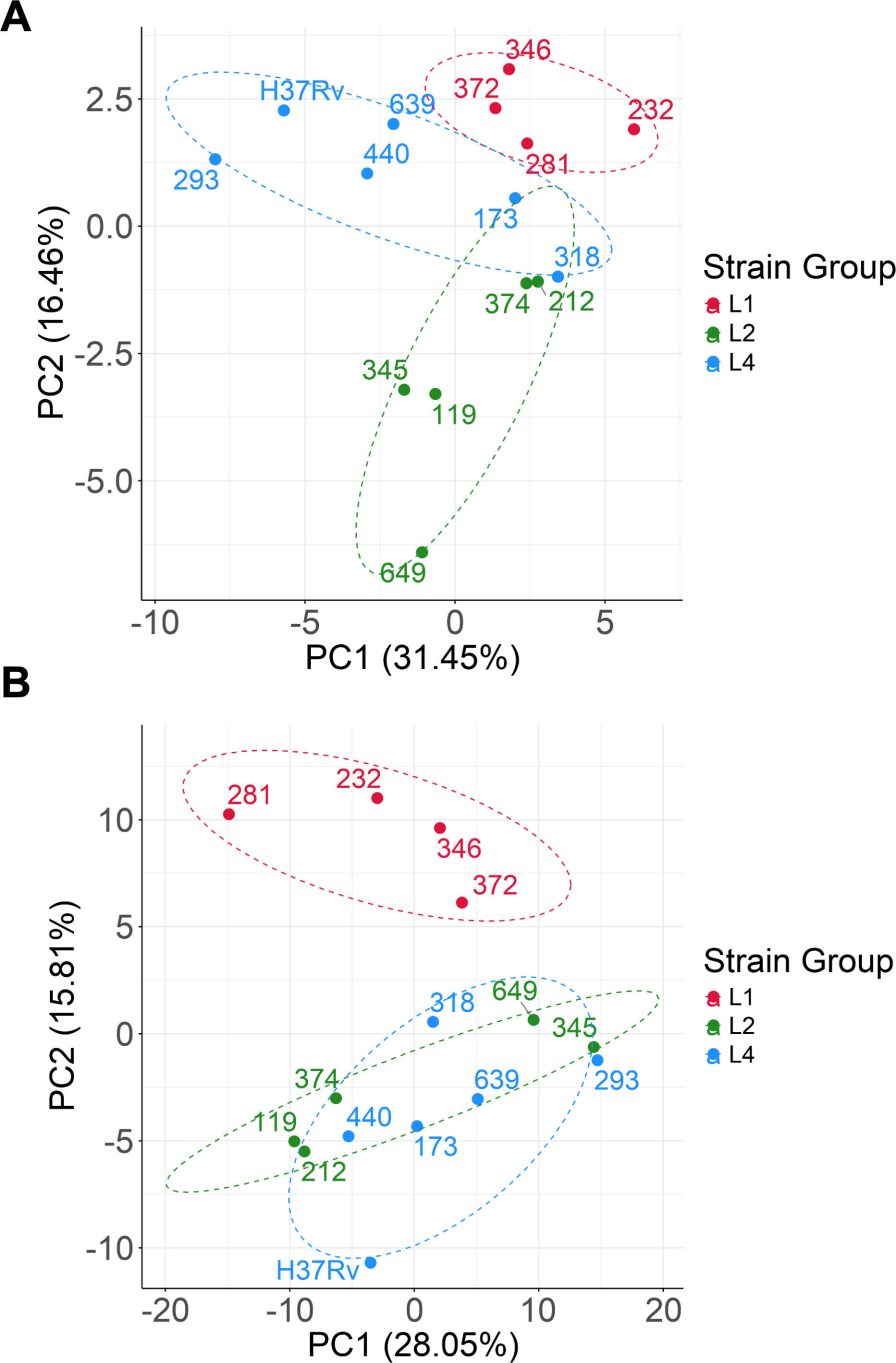
Principal component analysis of metabolomics data. (**A**) Targeted metabolomics data (43 metabolites). (**B**) Untargeted metabolomics data (266 metabolites). Dashed ellipses represent one standard deviation from the centroid (average position) of the group along each principal component. Figure made using prcomp and ggplot in R (v4.5.2).

As doubling time is only one aspect of bacterial growth, and our functional category analysis suggested significant metabolic remodeling in several pathways including cell wall metabolism, we carried out further untargeted and targeted metabolomics analysis using LC-MS on the intracellular metabolome of each strain. To compare the metabolomic profiles of each strain, we performed a principal component analysis of all targeted metabolites and used the data from Mtb H37Rv as a reference strain. This allowed us to determine the variability within and between lineage groups, for all 15 strains. Summarizing and plotting by the top two principal components, we found that each lineage had a distinct metabolomic profile, as defined by no overlap between the ellipses representing one standard deviation from the centroid of each group (Fig. 2 and Table S2).

When untargeted metabolites were included in the analysis, lineage 1 strains had a distinct metabolomic profile, according to the same definition, while lineages 2 and 4 strains had overlapping metabolomic profiles (Fig. 2B). These results indicate measurable differences in the metabolome of lineage 1 compared to lineages 2 and 4, as well as differences in certain metabolites between all three lineages. These larger metabolic differences between ancient lineage 1 and modern lineages 2 and 4 mirror the larger genetic changes between the ancient and modern lineages.

**Fig 3:**
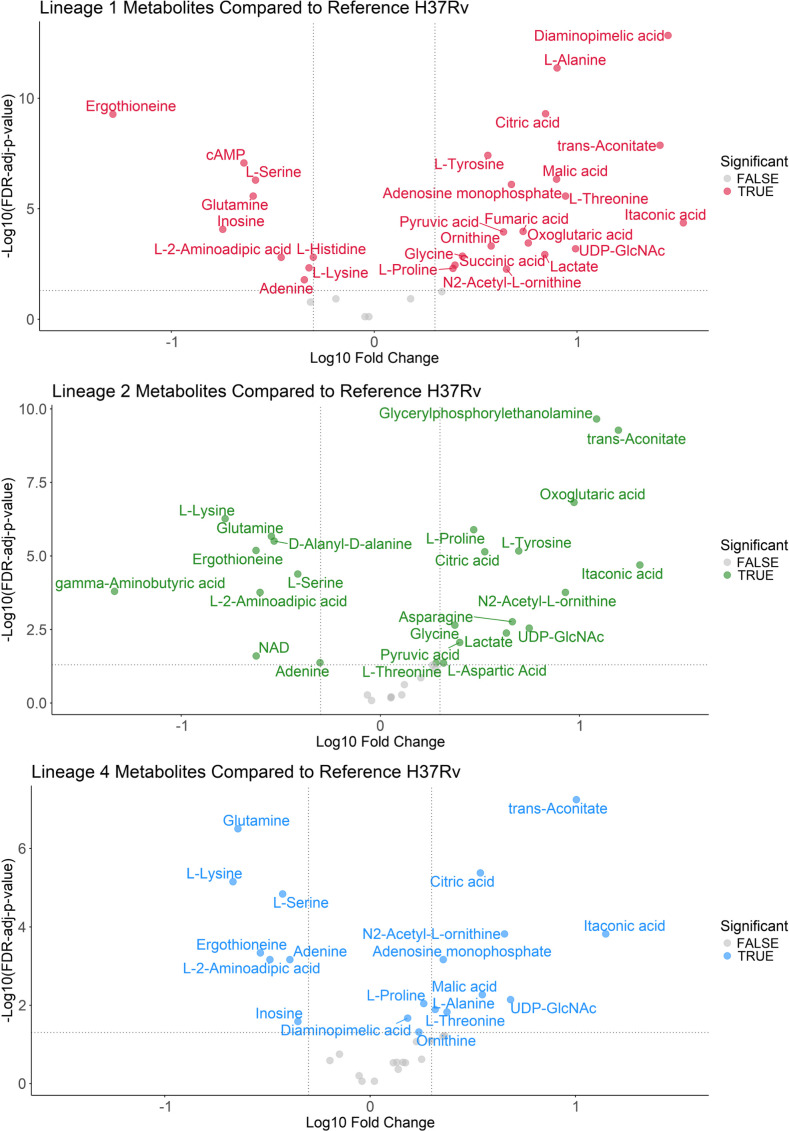
Lineage volcano plots of targeted metabolites with significant Log_10_FC. The Log_10_FC was calculated by subtracting the average reference H37Rv value from the average lineage value for each metabolite. Metabolites with an FDR-adjusted *P* value of <0.05 are colored (by lineage) and labeled. Each subplot shows a different lineage, 1, 2, or 4, with the label at the top. Figure made using tidyverse (v2.0.0) in R (4.5.2).

Comparison to the reference strain Mtb H37Rv allowed us to examine metabolic differences from the same baseline as genetic differences. Examining volcano plots of the metabolite fold changes from relative to H37Rv for each lineage helped identify the metabolites that drive the lineage-specific differences (Fig. 3). The largest unique changes were in metabolites such as NAD, ergothioneine, gamma-aminobutyric acid (GABA), glycerylphosphorylethanolamine (GPE), and diaminopimelic acid (DAP).

**Fig 4:**
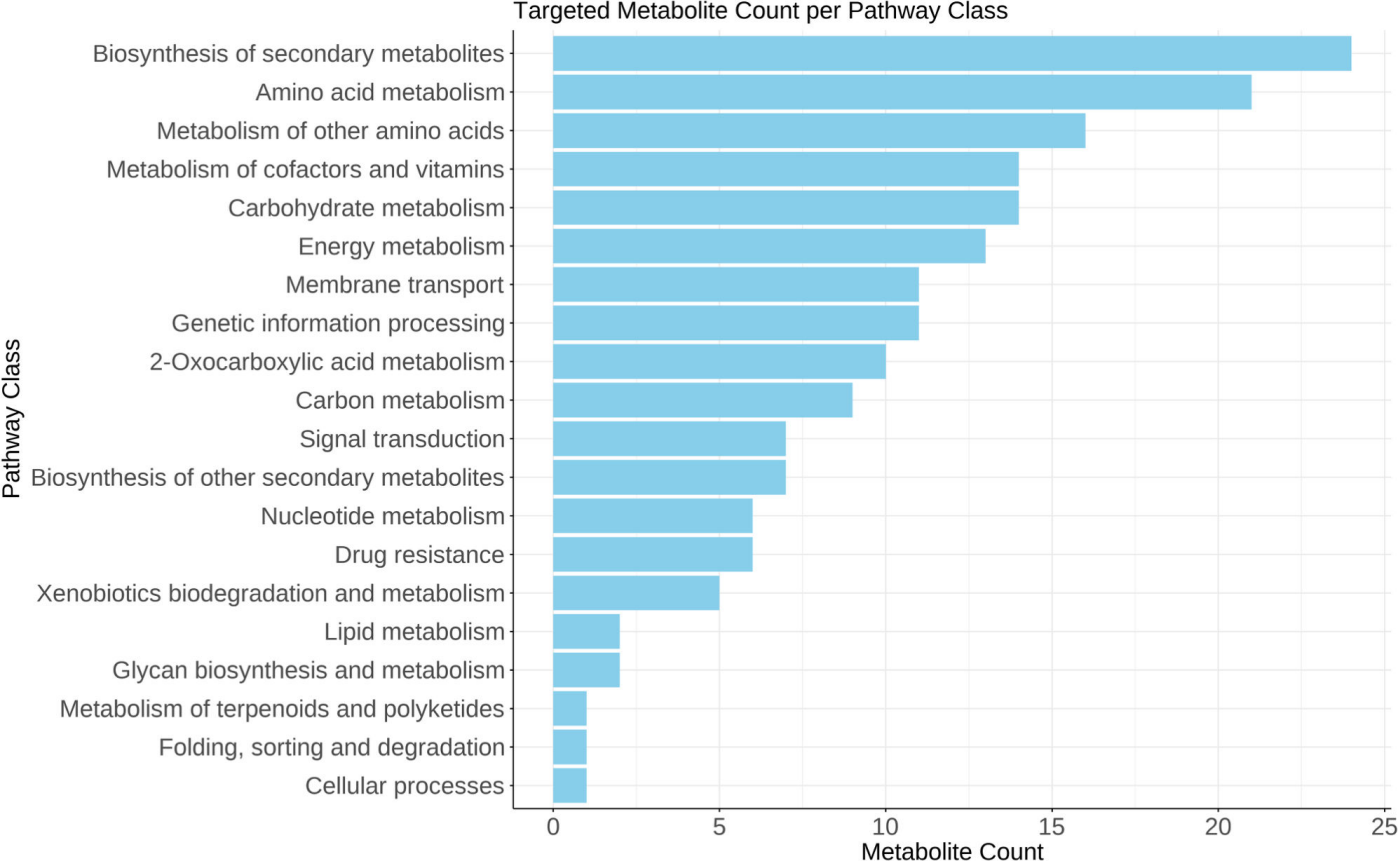
Bar plot of targeted metabolites assigned to Mtb KEGG pathway classes. The 43 targeted metabolites are categorized into pathway classes based on KEGG, with metabolites counted multiple times in each relevant class since many participate in multiple pathways. Figure made using tidyverse (v2.0.0) in R (4.5.2).

To better visualize pathway perturbation, each metabolite was classified using KEGG overall pathways, KEGG sub-pathways, and BioCyc or Rhea reactions (Fig. 4).

Most targeted metabolites fell into amino acid metabolism, metabolism of cofactors and vitamins, and carbohydrate metabolism. Similarly, most untargeted metabolites fell into amino acid metabolism, metabolism of cofactors and vitamins, and biosynthesis of secondary metabolites.

**Fig 5:**
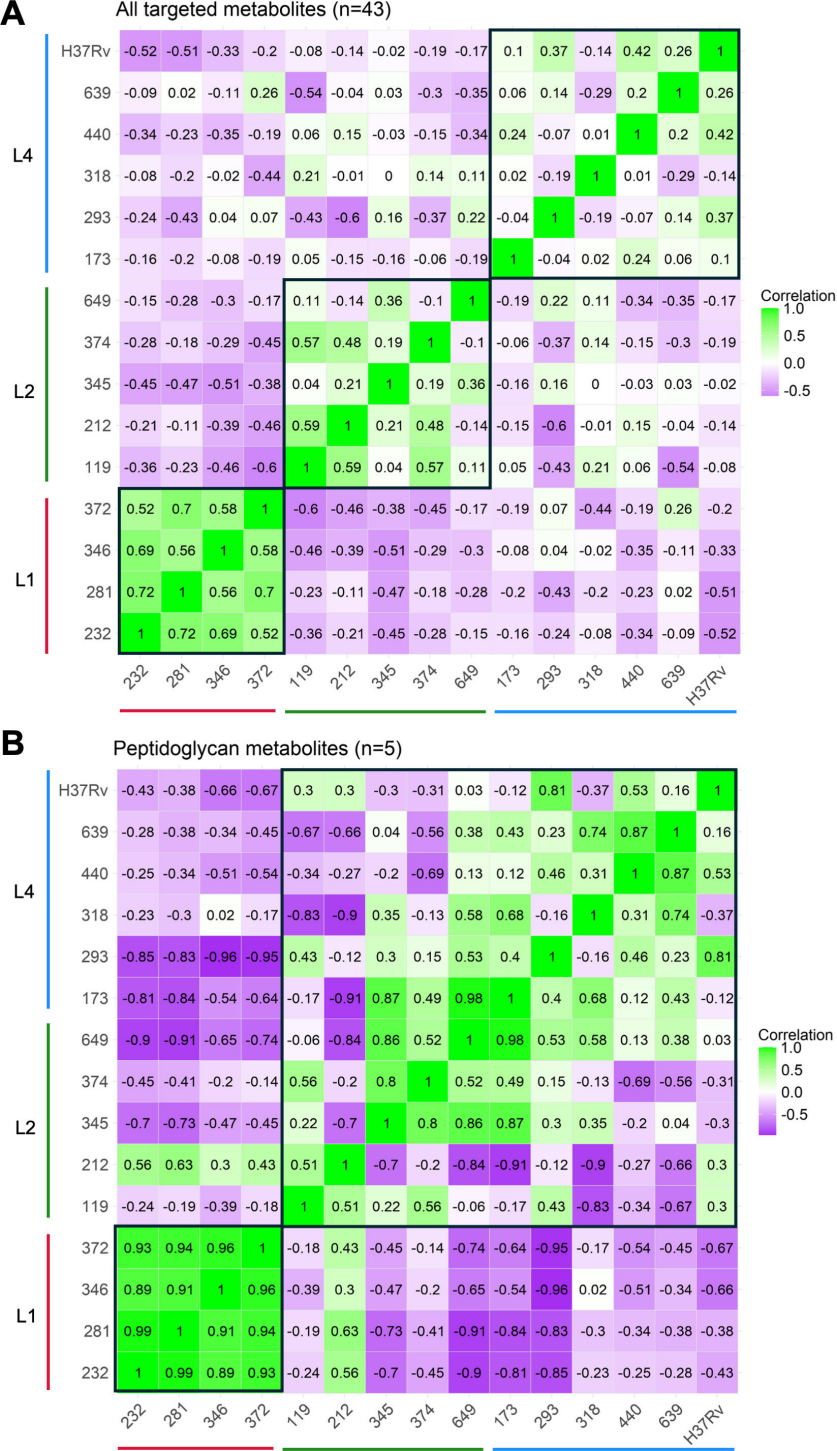
Pearson correlation plot of metabolites by strain. Each box indicates the correlation value between two strains, calculated by treating the metabolite values per strain as a vector with a Pearson correlation test. Strains are grouped by lineage (see the boxes), (**A**) includes all targeted metabolites (*n* = 43), and (**B**) only peptidoglycan biosynthesis metabolites (*n* = 5). Positive values are in green, and negative values in purple, with the midpoint at 0 as white. Note that the values used are not adjusted to the reference H37Rv, so that all strains are included and compared. Figure made using tidyverse (v2.0.0) in R (4.5.2).

**Fig 6:**
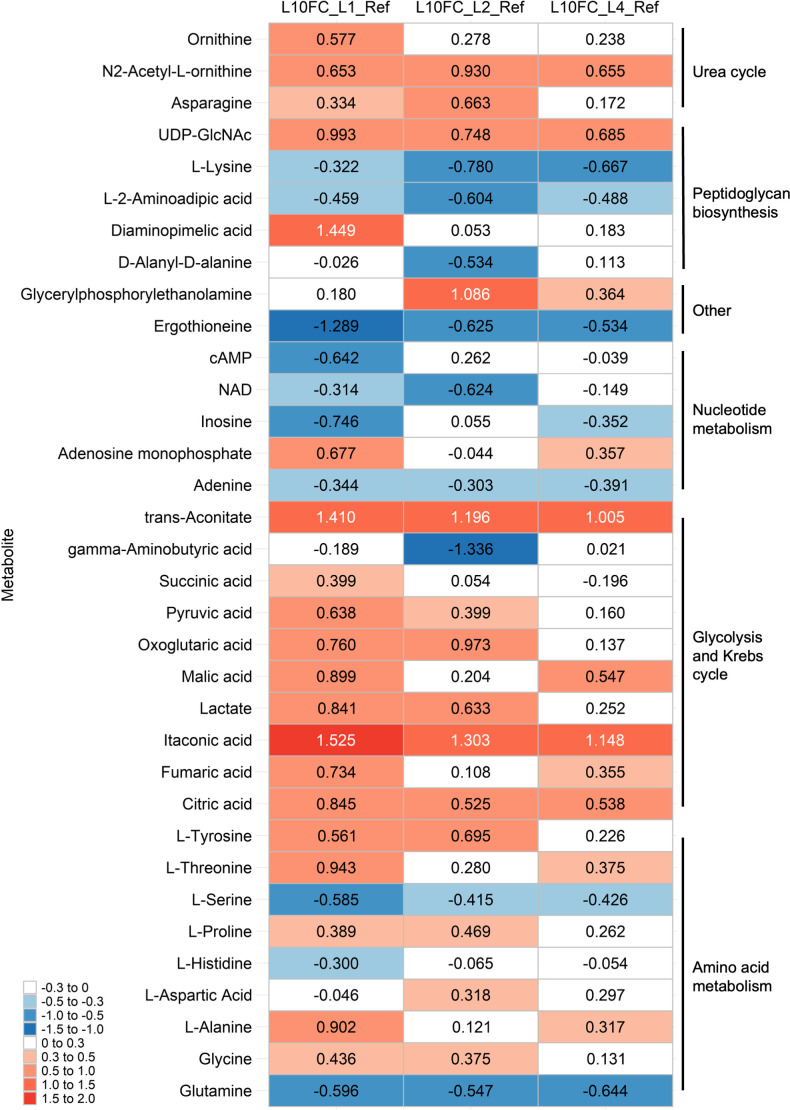
Heatmap of significant Log_10_FC for all targeted metabolites. Each column shows the average of the log_10_ scaled values for lineage strains compared to H37Rv. Each row is a metabolite included in the targeted analysis (the right panels show corresponding pathways). Each value is an L10FC (log_10_ fold change), in a color gradient where positive values are red, near 0 values are white, and negative values are blue. Significance was determined as described (≥0.3 L10FC and <0.05 FDR-adjusted *P* value). Figure made using tidyverse (v2.0.0) in R (4.5.2).

To examine the strain level differences and support lineage-specific metabolomic characterization, we performed a correlation analysis of the strains (Fig. 5). To illustrate the scale of the differences in key metabolites, we made a heatmap summarizing those with significant changes in targeted metabolite levels in at least one lineage compared to H37Rv (Fig. 6). The distinct metabolic regulation in each lineage is clearly shown by the color differences in the heatmap, and the differences are evident when comparing lineage 1 to lineages 2 and 4. The metabolites with the largest fold change differences are likely driving the distinct groupings in the PCA plot (Fig. 2A) and correlation plots (Fig. 5). Considering all targeted metabolites, strains in the same lineage are positively correlated, and strains in different lineages are negatively correlated. However, focusing on peptidoglycan biosynthesis targeted metabolites (*n* = 5), strains in either lineage 2 or lineage 4 are positively correlated, indicating this process is similar across these two lineages. We see significant changes in the peptidoglycan biosynthesis metabolites (l-lysine, aminoadipic acid, DAP, d-alanyl-d-alanine, and UDP-GlcNAc), nitrogen processing metabolites, including membrane lipid GPE, and redox regulator ergothioneine (and precursor histidine) between ancient lineage 1 and the modern lineages 2 and 4 (Fig. 6). In addition, the lineage 2 strains we examined had significantly higher levels of asparagine, aspartic acid, and GPE, and lower levels of aminobutyric acid and oxoglutaric acid, compared to lineages 1 and 4, including the reference H37Rv (Fig. 6).

## DISCUSSION

This study identified distinct metabolomic profiles between ancient lineage 1 and modern lineages 2 and 4. The largest lineage-specific distinctions were found for metabolites involved in peptidoglycan biosynthesis, redox homeostasis, and nitrogen metabolism. These stress response-related processes are tightly controlled by Mtb during infection and of particular interest in antibiotic development.

Previous work indicates that during infection, Mtb undergoes significant remodeling of the cell envelope to enhance its antibiotic tolerance and help its survival under the stressful environment inside the host (31). The metabolic changes we found in lineage 1, particularly the upregulated intracellular level of DAP, may indicate either impaired DAP incorporation into cell wall peptidoglycan or upregulated DAP synthesis (32). These changes could affect lipid homeostasis and lead to differences in lipid profiles, which are crucial during infection (33), and may alter host immune response (10, 34). Mtb cell wall has been targeted for therapeutic, for example, d-alanine analog d-cycloserine is an antibiotic that targets peptidoglycan biosynthesis and is mainly used to treat multi-drug-resistant Mtb (35). Meanwhile, lysine biosynthesis, for which DAP is a precursor, is also considered a promising Mtb drug target. Lineage-specific differences in these peptidoglycan synthesis metabolites could change the effectiveness of antibiotics targeting this pathway (36). Mtb is already known to undergo metabolic adaptations in the presence of stressors and certain antibiotics (37), and lineage-specific metabolic differences could further alter treatment response. Even for antibiotics with well-characterized drug targets, differences in metabolic regulation between lineages can change drug efficacies (38).

Similarly, ergothioneine is essential for Mtb stress tolerance in long-term infection and increases virulence and persistence (19, 20). Mtb maintains redox homeostasis via ergothioneine and mycothiol to prevent damage to biomolecules (20). Ergothioneine is a histidine derivative that acts as an essential antioxidant in Mtb, and its levels are mainly controlled by redox sensor proteins like WhiB3, through central carbon metabolism and lipid metabolism (20), and in late growth stages, by protein phosphorylation (39). Mutations in genes encoding essential proteins for ergothioneine biosynthesis result in increased reactive oxygen species and decreased antibiotic tolerance, showing how Mtb tightly controls redox regulation (20). We did not find mutations in genes known to influence ergothioneine levels (20), despite reduced levels of ergothioneine and histidine in lineage 1 compared to lineages 2 and 4. Levels of mycothiol, another redox metabolite, do not significantly differ between lineages, although previous work has shown links between mycothiol and ergothioneine levels (20, 40, 41). This suggests that lineage 1 may be at a disadvantage in an environment with high oxidative stress due to lower ergothioneine production. In this study, bacteria were cultured under low oxidative stress, and further tests comparing low and high redox stress conditions are needed to investigate whether lineage 1 would produce less redox components compared to lineages 2 and 4 when under stress and whether that would constitute a growth disadvantage.

Moreover, we found lineage-specific changes in nitrogen metabolism occurring along with mutations in nitrogen metabolism enzymes. Nitrogen assimilation by Mtb is necessary for growth and survival, through production of proteins and the release of ammonium to buffer the pH of the phagosome (42). Besides several significant changes in nitrogen metabolites for lineage 2 compared to lineages 1 and 4, we also noted a lineage 2 specific mutation (present in strains 119, 212, and 374) in the l-asparaginase enzyme AnsA (*rv*1538c). The mutation (G281S), while not in the binding site, is predicted by Missense 3D to cause structural strain, which could impact enzyme function and thus nitrogen processing in Mtb (43). Further work would be needed to confirm whether this mutation has any impact on enzyme function and Mtb growth, and to determine how widespread this mutation is by comparing a larger number of lineage 2 strains.

The main limitations of this work are the lack of gene expression information, the subset of metabolites, and limited gene annotations. Measuring gene expression levels would provide a link between the genome and metabolome, and could impact the results, but it is expensive and time-consuming at this scale. For example, our analysis could not fully explain the differences in carbohydrate metabolism. This is not unexpected, given the bacteria’s need to respond quickly to small changes in the environment and adapt to the stressful conditions in the phagosome; the transcriptome and interactions between metabolic pathways likely play a large role in metabolic regulation (44, 45). Illustrating this, we found decreased ergothioneine levels in lineage 1. Ergothioneine is thought to be regulated by redox sensor proteins and carbon and lipid metabolism (20, 39). We also found decreased histidine, an ergothioneine precursor, in lineage 1, but no change in mycothiol levels. Other large, unexplained changes in key metabolites may have similar regulation, despite the lack of genetic differences in the lineage. In addition, targeted metabolomics focuses on confidently identified compounds of known importance but leaves out many metabolites that would give better pathway coverage and which could link to enzymes of interest. Finally, many genes and metabolic pathways in Mtb have yet to be fully characterized, and this makes it difficult to predict the functional impact of SNPs in those genes.

In the future, this study could be extended by incorporating more strains and lineages to improve the identification of lineage-specific metabolic SNPs. Mtb sequences available in ENA could be aligned to Mtb H37Rv to determine whether the SNPs identified are truly present in most strains in one lineage. Second, to reveal how a reaction progresses in culture under various conditions, ^13^C isotope labeling can pinpoint shared carbon atoms after conversion into another molecule (46). Isotope labeling of metabolites clearly indicates which atoms are incorporated into another molecule during bacterial processing and provides evidence to pinpoint the details of connected metabolic processes and enzyme function (23, 30). Third, functional genetics approaches, such as the use of gene knock-in or knock-out, would be useful to compare growth and metabolism between lineages in further experiments to determine the impact of certain pathway perturbations on Mtb persistence. To put the culture conditions in context, the metabolic findings presented here are from bacteria cultured in a state resembling early host infection, with low nutrients and oxidative stress. Mid to late infection models and various stressors would provide a more complete picture of metabolic regulation over the Mtb life cycle. The mycobacteria could be cultured under various conditions (lacking nutrients, low oxygen, and low pH) to mimic the phagosome *in vitro* (46), in cultured phagosomes (47, 48), or in animal models of infection (49, 50). Overall, further studies would be valuable to determine the extent of metabolic differences between Mtb lineage groups.

In summary, this study reinforces the view that Mtb lineages possess considerable phenotypic diversity, highlights metabolic differences between ancient lineage 1 and modern lineages 2 and 4, and indicates that metabolomics profiling is a useful method to characterize lineage groups. These lineage-specific metabolomic adaptations have consequences in Mtb drug susceptibility, transmissibility, and virulence. Furthermore, comparing metabolic profiles across lineages could help evaluate the broad-spectrum potential of antibiotics targeting specific metabolic pathways in Mtb.

## Data Availability

Bash scripts and R programming language were used for the analysis described in this paper. The main metabolomics analysis code used can be found on the GitHub repository https://github.com/evoskoui/GENMETA-TB. The metabolomics data have been deposited to Metabolights repository (51) with the study identifier MTBLS14029.

## SUPPLEMENTAL MATERIAL

Table S1 spectrum.02887-25-s0001.xlsx List of metabolites from targeted list.

Table S2 spectrum.02887-25-s0002.xlsx Abundances of metabolites from targeted list.

Table S3 spectrum.02887-25-s0003.xlsx Normalized abundances of features from untargeted list.

ASM does not own the copyrights to Supplemental Material that may be linked to, or accessed through, an article. The authors have granted ASM a non-exclusive, world-wide license to publish the Supplemental Material files. Please contact the corresponding author directly for reuse.
